# Asbestos Burden Predicts Survival in Pleural Mesothelioma

**DOI:** 10.1289/ehp.11151

**Published:** 2008-02-12

**Authors:** Brock C. Christensen, John J. Godleski, Cora R. Roelofs, Jennifer L. Longacker, Raphael Bueno, David J. Sugarbaker, Carmen J. Marsit, Heather H. Nelson, Karl T. Kelsey

**Affiliations:** 1 Department of Environmental Health, Harvard School of Public Health, Boston, Massachusetts, USA; 2 Department of Pathology and Laboratory Medicine, Center for Environmental Health and Technology, Brown University, Providence, Rhode Island, USA; 3 Department of Pathology, Brigham and Women’s Hospital, Harvard Medical School, Boston, Massachusetts, USA; 4 Department of Work Environment, University of Massachusetts Lowell, Lowell, Massachusetts, USA; 5 Department of Environmental Health, Boston University School of Public Health, Boston, Massachusetts, USA; 6 Division of Thoracic Surgery, Brigham and Women’s Hospital, Harvard Medical School, Boston, Massachusetts, USA; 7 University of Minnesota School of Public Health, Division of Epidemiology and Community Health, Minneapolis, Minnesota, USA; 8 Department of Community Health, Center for Environmental Health and Technology, Brown University, Providence, Rhode Island, USA

**Keywords:** asbestos, mesothelioma, survival

## Abstract

**Background:**

Malignant pleural mesothelioma (MPM) is a rapidly fatal asbestos-associated malignancy with a median survival time of < 1 year following diagnosis. Treatment strategy is determined in part using known prognostic factors.

**Objective:**

The aim of this study was to examine the relationship between asbestos exposure and survival outcome in MPM in an effort to advance the understanding of the contribution of asbestos exposure to MPM prognosis.

**Methods:**

We studied incident cases of MPM patients enrolled through the International Mesothelioma Program at Brigham and Women’s Hospital in Boston, Massachusetts, using survival follow-up, self-reported asbestos exposure (*n* = 128), and a subset of cases (*n* = 80) with quantitative asbestos fiber burden measures.

**Results:**

Consistent with the established literature, we found independent, significant associations between male sex and reduced survival (*p* < 0.04), as well as between nonepithelioid tumor histology and reduced survival (*p* < 0.02). Although self-reported exposure to asbestos was not predictive of survival among our cases, stratifying quantitative asbestos fiber burden [number of asbestos bodies per gram of lung (wet weight)] into groups of low (0–99 asbestos bodies), moderate (100–1,099), and high fiber burden (> 1,099), suggested a survival duration association among these groups (*p* = 0.06). After adjusting for covariates in a Cox model, we found that patients with a low asbestos burden had a 3-fold elevated risk of death compared to patients with a moderate fiber burden [95% confidence interval (CI), 0.95–9.5; *p* = 0.06], and patients with a high asbestos burden had a 4.8-fold elevated risk of death (95% CI, 1.5–15.0; *p* < 0.01) versus those with moderate exposure.

**Conclusion:**

Our data suggest that patient survival is associated with asbestos fiber burden in MPM and is perhaps modified by susceptibility.

Malignant pleural mesothelioma (MPM) is a rapidly fatal malignancy with a median survival time of < 1 year. The single most important risk factor for MPM is exposure to asbestos, which occurs in ≥ 70–80% of these patients (Robinson and Lake 2005; Tammilehto et al. 1992). More than 3,000 deaths can be attributed to MPM each year in the United States, and worldwide its incidence is on the rise (Morinaga et al. 2001; Pelin et al. 1994; Price 1997; Price and Ware 2004; Roushdy-Hammady et al. 2001). As a result of the profound disease risk associated with exposure to asbestos and the occupational setting where exposure often occurs, litigation related to asbestos disease—estimated at $265 billion over the next 40 years—has become a tremendous economic burden (Bhagavatula et al. 2001). Although MPM incidence trends may plateau and begin to decline in the coming years in the United States (Price 1997; Price and Ware 2004), asbestos-containing products are still imported into the country. Moreover, asbestos use in other nations remains widespread and significant (Robinson and Lake 2005).

Following diagnosis of MPM, the options for treatment are in part dictated by known prognostic factors. Notable predictors of reduced survival in this disease are male sex and nonepithelioid histologies (Zellos and Christiani 2004). Recently, Flores et al. (2007) reported that a history of asbestos exposure is associated with reduced survival. In an effort to confirm and extend this observation, we used both self-reported (*n* = 128) and quantitative asbestos burden measures (*n* = 80) in a subset of cases to examine the relationship between asbestos exposure and MPM treatment outcome.

## Materials and Methods

### Study population and exposure data

Lung tissue and tumor tissue were obtained following surgical resection of pleural mesothelioma from incident cases seen at the International Mesothelioma Program at Brigham and Women’s Hospital during 2000–2006. All patients provided written informed consent under the approval of the appropriate institutional review boards.

Clinical information was obtained from review of each patients’ medical record. Pathologic diagnosis and date of diagnosis were obtained from the medical record of the initial diagnosis, either at Brigham and Women’s Hospital or the primary referring clinic, after having been confirmed by a pathologist’s review (J.G.G.). Each patient (*n* = 128) was assessed for history of exposure to asbestos by a trained industrial hygienist (83 cases had available asbestos body burden data), and additional demographic and environmental data on medical and occupational history was obtained through an in-person questionnaire or interview. Patients were followed up for survival using the National Death Index (National Center for Health Statistics 2007) to determine date of death. Surviving patients were censored (*n* = 48) based on their last known clinic visit.

We quantified asbestos bodies (ABs) in samples of lung tissue from multiple sites in the resected lung (De Vuyst et al. 1998); ABs per gram of lung (wet weight) were calculated as previously described (Churg and Warnock 1977).

### Statistical analysis

We performed univariate tests for association between asbestos exposure, asbestos body burden, patient demographic, and tumor characteristic data using SAS software (SAS Institute Inc., Cary, NC). Similarly, tests for association between these variables and survival were carried out with log-rank tests on Kaplan-Meier survival probability plot strata. Also, we used a Cox proportional hazards model to adjust for covariates when examining overall patient survival.

## Results

We obtained tumor and lung tissue from patients during surgical resection, and although surgically treated patients tend to be slightly younger and have more epithelioid disease compared to the total MPM patient population, this cohort is highly similar to other surgically treated cohorts (Pass et al. 2008). Survival data were available on all 128 cases; of these, 83 cases had available asbestos body burden data. Among the cases with available AB counts, three had extremely high counts—14,870, 19,681, and 303,852 ABs per gram of wet weight lung (ABs/g lung)—compared to the median count of 158. To avoid an analysis anchored by extreme values, we did not include data from these three patients.

In Table 1, we present exposure, demographic, and tumor histology data for all 128 cases and for the subset of 80 cases with asbestos burden data. Cases with asbestos burden data did not differ significantly from cases without fiber burden data.

**Figure 1:**
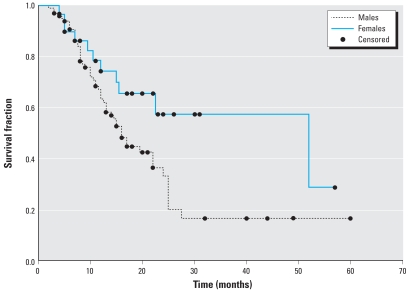
Kaplan-Meier survival probability plots of MPM patients (*n* = 128) by sex, using the log-rank method to test for a difference between strata. Males (*n* = 98) had significantly reduced survival compared to females (*n* = 30; *p* < 0.04). Surviving patients (*n* = 48) were censored.

**Figure 2:**
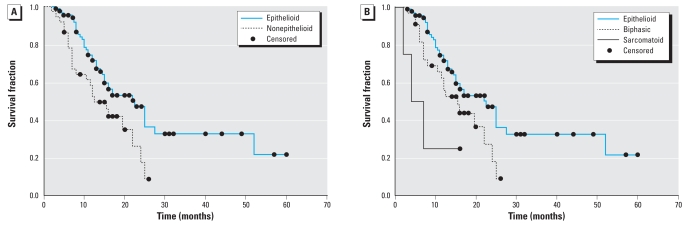
Kaplan-Meier survival probability plots of MPM patients (*n* = 128) based on tumor histology, using the log-rank method to test for a difference between strata. (*A*) Survival of patients with an epithelioid tumor (*n* = 91) and those with a mixed or sarcomatoid tumor (*n* = 37); patients with a nonepithelioid tumor had significantly reduced survival compared to those with an epithelioid tumor (*p* < 0.02; *n* = 128). (*B*) Survival of patients with an epithelioid (*n* = 91), biphasic (*n* = 33), or sarcomatoid tumor (*n* = 4); survival was significantly different among patients with the three tumor types (*p* < 0.01). Surviving patients (*n* = 48) were censored.

“Survival time” was defined as the time from diagnosis to death or last known follow-up. Figure 1 shows the Kaplan-Meier survival probability plots stratified by sex, and the log-rank test indicates a significantly reduced survival for males compared to females (*p* < 0.04). Similarly, Figure 2 shows the Kaplan-Meier survival plots by tumor histology. These data reveal a significant difference in survival between patients with epithelioid and nonepithelioid histologies (log-rank *p* < 0.02), as well as a significant difference among epithelioid, biphasic, and sarcomatoid histologies (log-rank *p* < 0.01).

**Figure 3:**
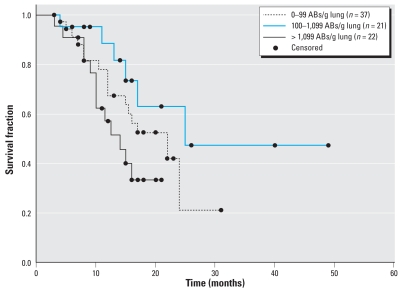
Kaplan-Meier survival probability plots of MPM patients based on asbestos burden, using the log-rank method to test for a difference among strata. Survival differences among exposure groups approaches statistical significance (*p* = 0.06). Eighty patients had AB counts; surviving patients (*n* = 48) were censored.

We then examined the relationships among asbestos exposure, asbestos fiber burden, patient demographics, tumor histology, and survival data, and we found a significant difference among asbestos fiber burden levels and survival. Among all 128 cases, self-reported exposure to asbestos was not predictive of survival in MPM (log-rank *p* = 0.44; data not shown). However, we observed a significant association between self-reported asbestos exposure and older age at diagnosis (reported exposure, 62.0 ± 9.5 years; no reported exposure, 56.9 ± 9.7 years; *t*-test, *p* < 0.05), as well as between male sex and reported asbestos exposure (Fisher’s, *p* < 0.0001; data not shown). Quantitative asbestos burden data from 80 cases showed that males (median count, 219 ABs/g lung; range*,* 0–6,211) had significantly higher asbestos burden than females (median count*,* 20 ABs/g lung; range*,* 0–2,437; Wilcoxon *p* < 0.0001). Models of survival by asbestos exposure did not demonstrate a linear trend; thus, data were stratified into tertiles for subsequent analysis. After stratifying asbestos burden data into tertiles of low burden (0–99 ABs/g lung), moderate burden (100–1,099 ABs/g lung), and high burden (> 1,099 ABs/g lung), we found an association of fiber burden with survival among these groups that approached statistical significance (Figure 3; log-rank *p* = 0.06). Using a Cox proportional hazards model to adjust for covariates, cases with low asbestos fiber burden had a 3-fold elevated risk of death [95% confidence interval (CI), 0.95–9.5; *p* = 0.06] compared to cases with moderate burden (Table 2). Patients with high asbestos fiber burden had 4.8-fold elevated risk of death (95% CI, 1.5–15.0; *p* < 0.01) compared to patients with moderate burden (Table 2). Including the three cases with extreme outlying asbestos counts in this model did not significantly alter the results (data not shown).

## Discussion

In this study we evaluated the relationships among asbestos exposure, asbestos fiber burden, patient demographics, tumor histology, and survival in MPM. Similar to other groups, we found that male sex and nonepithelioid histologies predict reduced survival (Flores et al. 2007). Interestingly, we also demonstrated that after correcting for covariates, low or high lung tissue asbestos burden predicted a higher risk of death compared to moderate asbestos burden.

Historically, most asbestos exposure is occupationally related and affects individuals who mined, manufactured, or applied asbestos-containing products (McDonald and McDonald 1980). Given that men are more likely to be employed in asbestos-associated occupations, it is not surprising that they have higher levels of fiber burden and that the ratio of men to women with MPM is between 3:1 and 5:1 (Zellos and Christiani 2004). Our case series follows this pattern: Men with MPM have a significantly higher lung tissue asbestos burden and outnumber women more than three to one. Men have both higher fiber burdens and significantly reduced survival compared to women, making it reasonable to posit that an increased asbestos fiber burden may contribute to poor survival per se. Consistent with this, we observed an increased risk of death among patients with high asbestos burden compared to patients with moderate asbestos burden. However, we also found an increased risk of death among patients with the lowest lung tissue asbestos burden versus those with moderate fiber burden.

The mechanism responsible for this unusual dose–response association with survival is unclear. One possibility is that cases with low asbestos burden were exposed to chrysotile asbestos or other naturally occurring mineral fibers, such as erionite, that have been associated with MPM (Carbone et al. 2002). Chrysotile asbestos is less biopersistent and is considered by many to be less pathogenic than amphibole asbestos. Hence, significant exposure to chrysotile could have occurred in those with lower numbers of ABs, and this might not be evident in our data. However, lung chrysotile fiber burden has been shown to correlate with asbestos body levels, arguing against significant chrysotile exposure (Butnor et al. 2003).

Concomitantly, erionite fibers are reported to have the highest carcinogenic potential of studied fibers, and form ferruginous bodies indistinguishable from ABs (Dumortier et al. 2001). Because erionite fibers do not form ferruginous bodies as readily as asbestos fibers, the AB counts in individuals with erionite exposure may underestimate their true internal dose (Dumortier et al. 2001). The worldwide geographic distribution of erionite is very limited; therefore, it is unlikely that patients in this study had this exposure. However, if either of these scenarios were true (patients with low AB counts having significant chrysotile or erionite exposure), it would imply that the fiber dose is directly associated with survival.

A more likely explanation of our results is related to the considerable literature that has documented both the absence of an appreciable threshold for asbestos-induced mesothelioma, and the fact that MPM can occur with very low-level exposures (Hansen et al. 1998; Hodgson and Darnton 2000). Further, widespread exposures to asbestos, particularly environmental exposures in some parts of the world, combined with the rare incidence of mesothelioma, suggest that there may be susceptible individuals. In fact, multiple reports indicate that genetics may modify susceptibility to MPM (Ascoli et al. 1998; Dawson et al. 1992; Dogan et al. 2006; Hammar et al. 1989; Li et al. 1978; Lynch et al. 1985; Martensson et al. 1984; Otte et al. 1990; Precerutti et al. 1990; Risberg et al. 1980). When closely examining our asbestos fiber burden data, we found that most of the cases within the low-asbestos-burden group (0–99 ABs/g lung) had AB counts within the general population mean of 0–20 ABs/g lung (Dodson and Atkinson 2006); thus these patients may have a greater inherent susceptibility. Further, our data lead to the hypothesis that patients with high susceptibility suffer from more aggressive disease. Outside this high susceptibility group, the other two tertiles demonstrated a dose–response relationship between asbestos fiber burden and survival.

In summary, our data suggest that patient survival is associated with asbestos fiber burden in pleural mesothelioma and that this association is perhaps modified by susceptibility. Studies using larger case groups—ideally with chrysotile and erionite exposure data—are necessary to further elucidate the ability of asbestos burden to predict survival in MPM.

## Figures and Tables

**Table 1:** Demographics and tumor characteristics of mesothelioma patients.

Characteristic	Total (*n* = 128)	Asbestos burden data (*n* = 80)
Sex		
Female	30 (23)	20 (25)
Male	98 (77)	60 (75)
Age (years)		
Range	30–85	30–80
Mean ± SD	62 ± 10.1	61 ± 9.8
Histology		
Epithelioid	91 (71)	60 (75)
Mixed	33 (26)	18 (22.5)
Sarcomatoid	4 (3)	2 (2.5)
Asbestos exposure^a^		
Yes	95 (74)	59 (74)
No	33 (26)	21 (26)
AB count (ABs/g lung)^b^		
Range (median)	NA	0–6,211 (128)
Mean ± SD	NA	875 ± 1,467

NA, not applicable. Values are number (%) except where indicated.

a Self-reported.

b Data for 83 cases available; three outliers removed.

**Table 2:** Asbestos body burden predicts survival in MPM, Cox’s proportional hazards model.

Covariate	No. (%)	Hazard ratio (95% CI)	*p*-Value
Sex			
Male	60 (75)	1.0 (reference)	
Female	20 (25)	0.72 (0.27–1.9)	0.94
Histology			
Epithelioid	54 (77)	1.0 (reference)	
Mixed	14 (20)	0.82 (0.38–1.8)	0.62
Sarcomatoid	2 (3)	3.7 (0.35–39.1)	0.28
AB count (ABs/g lung)			
0–99	37 (46)	3.0 (0.95–9.5)	0.06
100–1,099	21 (26)	1.0 (reference)	
> 1,099	22 (28)	4.8 (1.5–15.0)	<0.01

The model controlled for age and all variables shown.
